# Supplementary figures and images for: Uncoupling of DNA Replication and Centrosome Duplication Cycles Is a Primary Cause of Haploid Instability in Mammalian Somatic Cells
Source: Front Cell Dev Biol. 2020 Jul 30;8:721. doi: 10.3389/fcell.2020.00721 (PMC7408703; doi:10.3389/fcell.2020.00721)

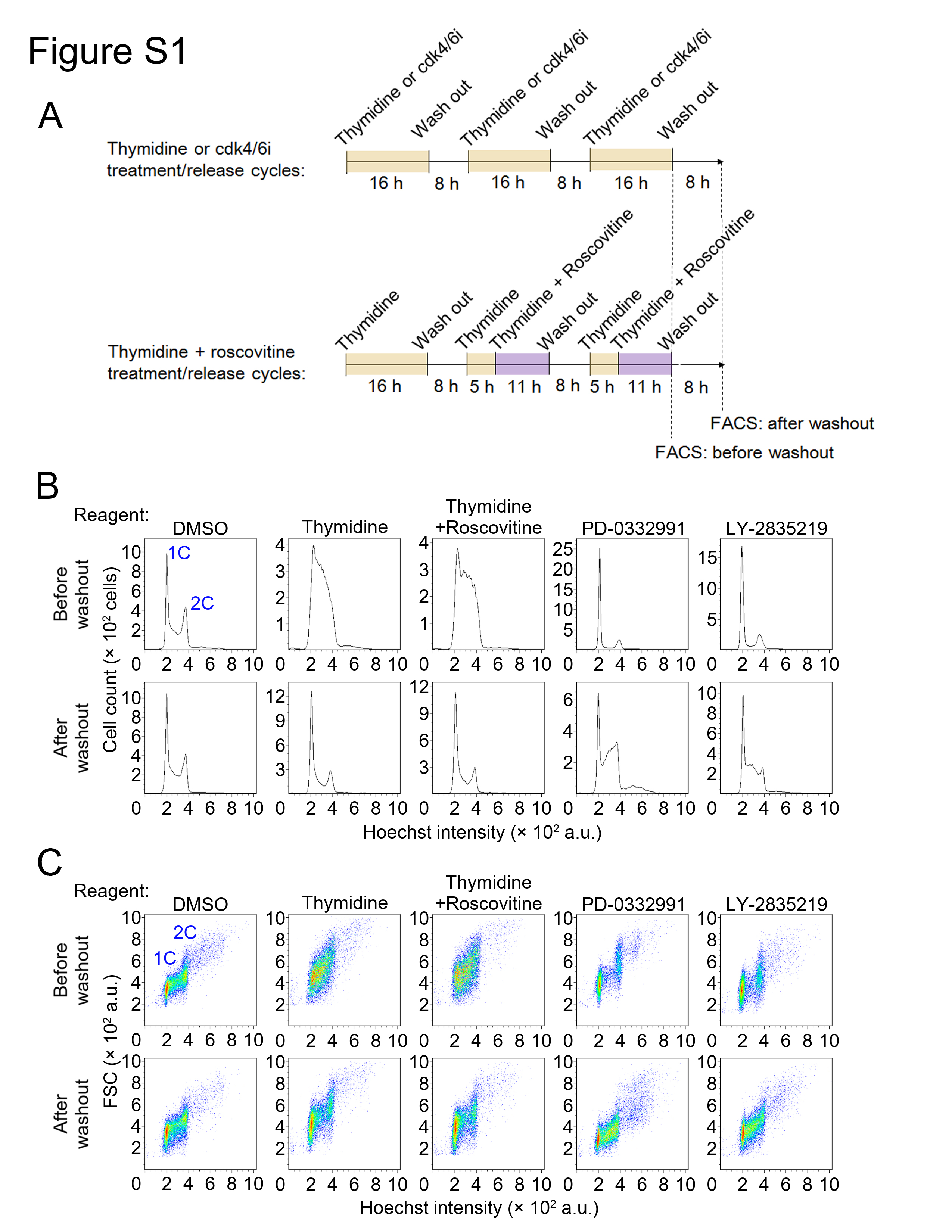

Supplement: FIGURE S1 — Intermittent extension of DNA replication cycle by cell cycle inhibitor treatment/release. (A) Experimental scheme of inhibitor treatment/release cycles. (B,C) Flow cytometric analysis of DNA content in Hoechst-stained haploid cells in inhibitors treatment/release cycles. Histograms of Hoechst signal and dot plots of forward scatter signal (for the judgment of relative cell size) against the Hoechst signal are shown in panels (B,C), respectively. Representative data from three independent experiments. Note that co-treatment of roscovitine did not change the profile of cell cycle arrest/release in the thymidine treatment/release cycle. (D,E) Timecourse flow cytometric analysis of DNA content (Hoechst signal; D,E) and PI incorporation (E) in haploid cells after the first washout of thymidine. (F) Proportions of PI-positive cells or sub-G1 population in panel (E). Means ± SE of three independent experiments (∗p < 0.05, two-tailed t-test). [file Image_1.jpeg]

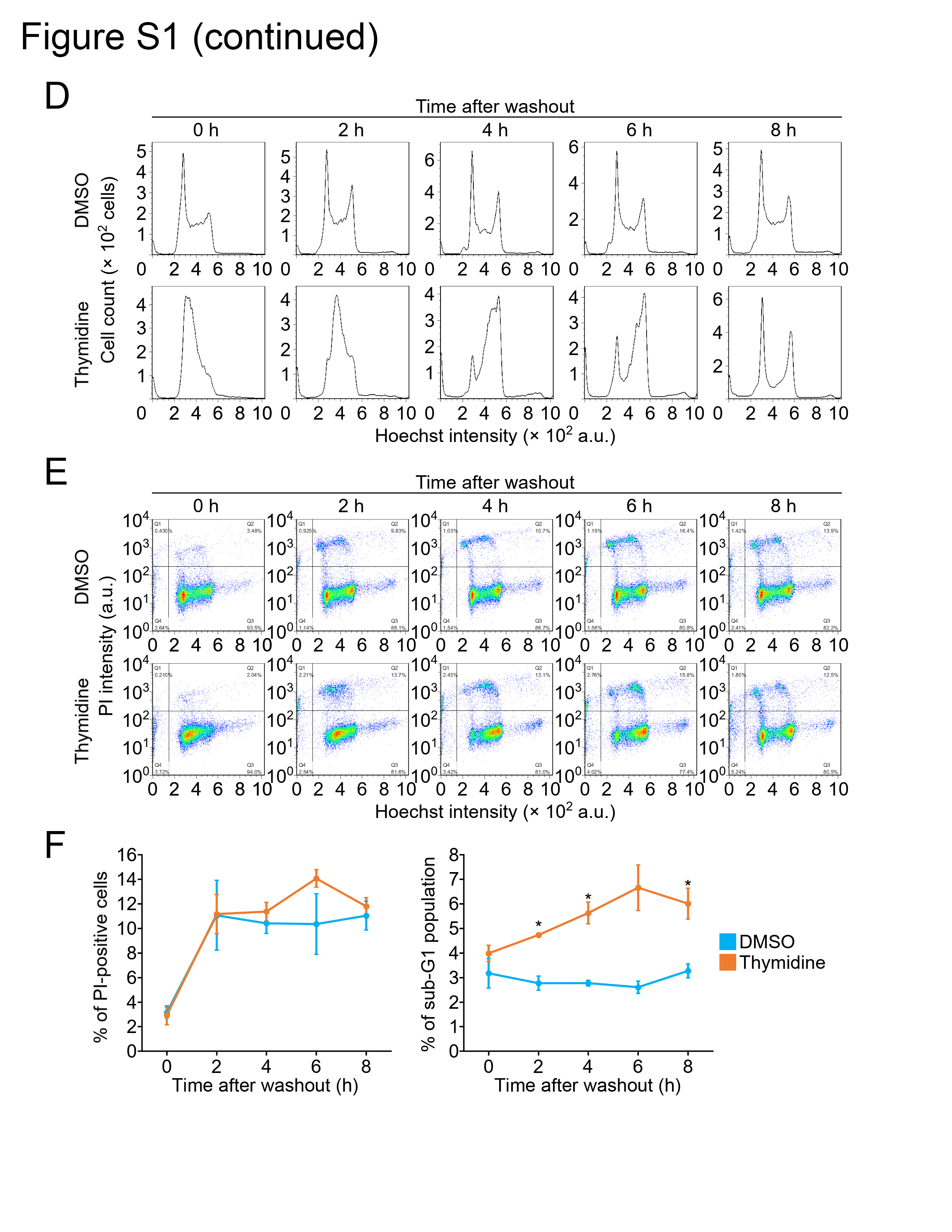

Supplement: Supplementary file 2 [file Image_2.jpeg]
