# Supplementary material for: Uncoupling of DNA Replication and Centrosome Duplication Cycles Is a Primary Cause of Haploid Instability in Mammalian Somatic Cells
Source: Front Cell Dev Biol. 2020 Jul 30;8:721. doi: 10.3389/fcell.2020.00721 (PMC7408703; doi:10.3389/fcell.2020.00721)
Supplement: Supplementary file 3 [file Table_1.DOCX]

Table S1: Parameters and constants used in the simulations

| Constants  （Unit） | Meaning | No mitotic restoration,  no cell cycle arrest  (untreated control) | No mitotic rescue,  24-h intermittent cell cycle arrest  (Virtual condition) | Mitotic restoration,  24-h intermittent cell cycle arrest  (thymidine-treated sample) |
| --- | --- | --- | --- | --- |
| $N_{haploid}(0)$  ($\times$10^3^ cells) | The initial number of haploid cells | 5 | 5 | 5 |
| $N_{diploid}(0)$  ($\times$10^3^ cells) | The initial number of diploid cells | 0 | 0 | 0 |
| *q_slippage_*  (h) | Incidence rate of mitotic slippage per mitotic event | 0.012 | 0.012 | 0.001 |
| *q_death_*  (h) | Incidence rate of mitotic death per mitotic event | 0.010 | 0.010 | 0.007 |
| τ_haploid_  (h) | Cell cycle length of haploid | 13.4 | 24 | 24 |
| τ_diploid_  (h) | Cell cycle length of diploid | 11.9 | 24 | 24 |
| *F_haploid, G1_*  (%) | G1 cell fraction within haploid population | 50 | 50 | 50 |
